# Neural correlates of executive dysfunction in alcohol use disorder: preliminary evidence from ^18^F-FDG-PET

**DOI:** 10.3389/fpsyg.2025.1568085

**Published:** 2025-05-12

**Authors:** Maria Arioli, Irene Bossert, Daniela D’Ambrosio, Marina Manera, Elena Maria Andreolli, Nicola Canessa, Giuseppe Trifirò

**Affiliations:** ^1^Department of Human and Social Sciences, University of Bergamo, Bergamo, Italy; ^2^Istituti Clinici Scientifici Maugeri IRCCS, Nuclear Medicine Unit of Pavia Institute, Pavia, Italy; ^3^Istituti Clinici Scientifici Maugeri IRCCS, Medical Physics Unit of Pavia Institute, Pavia, Italy; ^4^Istituti Clinici Scientifici Maugeri IRCCS, Clinical Psychology Unit of Pavia Institute, Pavia, Italy; ^5^IUSS Cognitive Neuroscience (ICoN) Center, Scuola Universitaria Superiore IUSS, Pavia, Italy; ^6^Istituti Clinici Scientifici Maugeri IRCCS, Cognitive Neuroscience Laboratory of Pavia Institute, Pavia, Italy

**Keywords:** PET, alcohol use disorder, neuropsychology, executive function, salience network, intervention, working-memory, ageing

## Abstract

Neuroimaging studies have shown that cognitive impairments in Alcohol Use Disorder (AUD), particularly involving executive functions, reflect widespread structural and functional brain alterations. However, these findings mostly result from magnetic resonance imaging (MRI). To complement previous MRI findings with a more direct measure of brain metabolism, we therefore explored the neural bases of executive impairments in AUD using FDG-PET. Twenty-three AUD patients and 18 healthy controls underwent a neurocognitive assessment, and patients also an ^18^F-FDG-PET scan. Using as reference for brain metabolism a FDG-PET dataset of age-matched healthy controls, we assessed a relationship between executive impairment and regional hypometabolism in AUD patients, while also considering a possible moderating age effect. Compared with controls, AUD patients exhibited widespread hypometabolism in the anterior/midcingulate cortex, fronto-insular cortex, and medial precuneus, supporting the hypothesis that their impaired executive performance might reflect an altered transition from automatic to controlled processing. Patients’ worse executive performance reflected in higher metabolism in the midcingulate cortex and medial precuneus, suggesting a possible compensatory neural mechanism. This relationship was moderated by age in the right anterior insula, where the decrease of metabolism is steeper, in older patients, at the lowest level of cognitive performance. This finding suggests that an age-related decrease in the compensatory capacity of the insular node of the salience network might contribute to cognitive decline in older patients. While supporting the use of FDG-PET to improve the understanding of AUD-related cognitive decline, and differential diagnosis in older patients, these findings might help design personalized innovative treatment protocols.

## 1 Introduction

Alcohol use disorder (AUD) is a chronic clinical condition marked by a compulsive intake of alcohol despite its adverse physical, psychological, and social effects, leading to approximately 3.3 million deaths annually (WHO, 2014). The burden of these effects is enhanced by AUD patients’ *cognitive* impairments (Wang et al., 2023), particularly involving executive functions (Chanraud et al., 2007; Jang et al., 2007) which play a key role in difficulties concerning impulse control, decision-making and goal-directed behavior (Alarcon et al., 2015; Bickel et al., 2017; Green et al., 2010; Pitel et al., 2009; Ramey and Regier, 2019).

The frequent pattern of executive impairment observed in AUD (Trick et al., 2014; Wiers et al., 2015; see Maharjan et al., 2022) has been initially ascribed to the specific susceptibility of the frontal cortex (i.e., “*Frontal lobe hypothesis*”; Dao-Castellana et al., 1998; Oscar-Berman and Marinkovic, 2003; Uekermann et al., 2003). However, the growth of multimodal neuroimaging techniques—and particularly functional magnetic resonance imaging (*f*MRI)—unveiled a more extensive pattern of brain damage in AUD, prompting an alternative “*Diffuse Brain Hypothesis*” (Stavro et al., 2013). More recent evidence rather highlights the role played by *distinct specific* neural systems, including fronto-insular, cingulate and parietal regions (Fama et al., 2019). This view has been strengthened by Voxel-Based Morphometry (VBM) evidence of grey matter (GM) atrophy not only in frontal structures such as lateral prefrontal cortex, anterior cingulate and insular-opercular cortex, but also in the posterior cingulate cortex alongside hippocampus, thalamus and striatum (Xiao et al., 2015; Yang et al., 2016). This pattern of cortico-striatal-thalamic structural damage (Peters et al., 2016; see Cushnie et al., 2023) supported the hypothesis that, in AUD, the well-known impairment of attentional and cognitive control might reflect structural and/or functional alterations in the so-called *salience network* underlying the switch from automatic to controlled processing (Butcher et al., 2022; Grodin et al., 2017; Menon and Uddin, 2010; Padula et al., 2022).

Increasing evidence indeed shows that this switch entails the transition between two anti-correlated networks, i.e., from the default mode network (DMN) to the executive control network (Fox and Raichle, 2007), when behaviorally relevant stimuli are detected by the fronto-insular and dorsal anterior cingulate (dACC) nodes of the salience network (Goulden et al., 2014; Menon and Uddin, 2010). The hypothesis of an impaired switch from automatic to controlled processing in AUD patients—contributing to their executive deficits—is nowadays supported by considerable structural and functional evidence. On the hand, GM atrophy in the insular and dACC sectors of the salience network has been shown to explain about half of variability in the attentional and working-memory impairment among AUD patients (Galandra et al., 2018). This structural evidence has been complemented by resting-state *f*MRI (rs-*f*MRI) studies, unveiling differences, across AUD patients and healthy individuals, in multiple facets of the intrinsic brain functional architecture, and in its relationship with behavioral performance (Canessa et al., 2022; Galandra et al., 2019). First, rs-*f*MRI studies have provided evidence of abnormal intrinsic activity in the DMN in AUD patients (Chanraud et al., 2011, 2013; Fede et al., 2019; Song et al., 2021). Moreover, they unveiled a relationship between executive impairments and altered intrinsic connectivity within and between resting-state networks underlying reward processing (Camchong et al., 2013; Maleki et al., 2022; Müller-Oehring et al., 2013, 2015), salience detection (Sullivan et al., 2013; Zhu et al., 2017) and the engagement of executive control (Weiland et al., 2014; Zhu et al., 2017). Via diffusion-tensor imaging (DTI), we found that these neuro-structural and functional correlates of AUD patients’ executive impairment represent different facets of a common neuropathological process involving alterations of white matter (WM) structural connections among salience, executive and default mode networks (Crespi et al., 2020). Overall, these findings appear to support the notion that cognitive impairments are at least partially explained by disrupted functional dynamics among large-scale neural networks in AUD (Crespi et al., 2019; see Spindler et al., 2022).

This multifaceted evidence shows that (*f*)MRI studies have significantly advanced our understanding of the neural bases of cognitive impairment in AUD. However, further valuable insights into this issue might come from other neuroimaging techniques, and particularly ^18^F-Fluoro-Deoxy-Glucose Positron Emission Tomography (FDG-PET). By tracking glucose metabolism, FDG-PET provides a direct assessment of regional brain activity, its changes in pathological conditions (Bralet et al., 2022), and whether/how they relate to cognitive-behavioral alterations (Clergue-Duval et al., 2020; Segobin et al., 2015). Differences in regional cerebral blood flow (CBF) across AUD and healthy controls should be therefore assessed as a potential confound of activation patterns in task-related or resting-state *f*MRI studies (Sullivan et al., 2021). FDG-PET might therefore provide a truly “functional” perspective on the relationship between neural and cognitive alterations in AUD, thereby complementing and enriching the widespread structural (Hahn et al., 2022) and functional (Taebi et al., 2022) evidence resulting from (f)MRI studies. Moreover, FDG-PET plays a key role in the early and differential diagnosis of neurodegenerative diseases (Guillén et al., 2020; Nestor et al., 2018), and in predicting the progression from subjective or mild cognitive impairment to dementia (Cotta Ramusino et al., 2024), as well as cognitive decline in patients without dementia (Torosyan et al., 2017). Nevertheless, neuroimaging studies on AUD are mostly based on MRI. With few exceptions (e.g., Clergue-Duval et al., 2020; Ritz et al., 2019), PET has been mainly focused on metabolic rates and dopaminergic transmission without assessing the associated cognitive deficits (e.g., Bralet et al., 2022; Ritz et al., 2016; see Spitta et al., 2023; Volkow et al., 2017), and on Korsakoff syndrome (KS), i.e., an alcohol-induced neurocognitive disorder characterized by severe anterograde amnesia that can persist chronically (Maillard et al., 2021; Pitel et al., 2009). These studies reported hypermetabolism in the cerebellum, and hypometabolism in fronto-insular and middle cingulate cortex, in KS (Maillard et al., 2021; Pitel et al., 2009), as well as cerebellar hypermetabolism, related to ataxia and working-memory deficits, in AUD (Ritz et al., 2019).

Combining neuropsychological, MRI and FDG-PET evidence might therefore increase the potential of neuroimaging data to characterize the neuro-functional changes underlying specific cognitive impairments in AUD, thereby supporting differential diagnosis in older patients, the development of targeted treatment protocols, and the assessment of their effectiveness. Importantly, pursuing this goal entails investigating also whether the relationship between regional brain metabolism and executive performance is itself modulated by patients’ age (Ewers et al., 2014). Building on previous findings from MRI studies, and to provide a more direct assessment of brain metabolism, the present study investigates the neural bases of cognitive-executive impairment in AUD through FDG-PET. Based on our previous VBM (Galandra et al., 2018) and rs-*f*MRI (Canessa et al., 2022) findings, we predicted that the degree of AUD patients’ executive impairment, and its relationship with their age, would reflect decreased metabolism, compared with age-matched healthy individuals, in fronto-insular and/or posterior frontomedial structures in charge of salience detection.

## 2 Materials and methods

### 2.1 Participants

The study involved 23 AUD patients (9 females; mean age: 45.70 years, standard deviation (SD): 7.82; mean educational level: 10.00 years; SD: 2.63) and 18 healthy control (henceforth “HCs”) participants (8 females, mean age, 44.83 years, SD, 8.86, mean educational level, 10.11 years, SD: 2.78). There were no significant group differences concerning age, education level, gender distribution or smoking status (Table 1). Patients joined the study after completing a 28-days in-patient alcohol withdrawal treatment at the Functional Rehabilitation Unit of ICS Maugeri-Pavia (Italy), and following at least 10 days of detoxification via standard medical treatments (benzodiazepine treatment was stopped at least 8 days before PET scanning). We assessed participants’ drinking history (i.e., quantity, type, and duration of alcohol use), and measured alcohol consumption as the average number of standard units of alcohol (UA) per day (with 1 UA corresponding to 12 g of ethanol, i.e., approximately 330 mL of beer, 125 mL of wine, or 40 mL of hard liquor). In patients, disease duration varied from 1 to 26 years (mean: 10.8 years ± 7.21). Inclusion criteria for patients were: (a) 20 < age<65 years; (b) AUD diagnosis based on DSM-5 criteria. Exclusion criteria for both groups included: presence/history of neurological/psychiatric disorders other than AUD (with the exception of nicotine dependence), current use of psychotropic substances/medications, past brain injury or loss of consciousness, severe medical conditions, inability to complete the neuropsychological assessment, and contraindications to PET. HCs were excluded in case of history of alcohol abuse based on a threshold of average alcohol consumption <2 UA for males and 1 UA for females. HCs were asked to remain abstinent from alcohol for at least 10 days prior to the study, with compliance verified through pre-experiment interviews. All participants provided their written informed consent to the study protocol, that was designed according to the latest version of the Declaration of Helsinki and approved by the ICS Maugeri Ethical Committee (Pavia, Italy).

**Table 1:** Demographics and alcohol use variables.

Group comparisons							
	Mean HC (*n* = 18)	Mean AUD (*n* = 23)	SD HC	SD AUD	DF	T-score	*p*-value
Demographic variables							
Age (years)	44.83	45.69	8.86	7.82	39	−0.33	0.371
Education level (years)	10.11	10.0000	2.78	2.63	39	0.13	0.448
Gender (female/total)	8/18	9/23					0.987
Smoking status (yes/total)	7/18	16/23					0.100

Alcohol use variables	Mean all patients	SD all patients	Mean females	SD females	Mean males	SD males	*p*-value
Duration of alcohol use (years)	10.80	7.21	11.89	7.11	10.11	7.48	0.576
Average daily alcohol dose (UA)	14.48	6.55	14.94	5.92	14.18	7.12	0.791

The upper section of the table presents the demographic variables for the AUD and HC groups, as well as the outcomes of group comparisons based on two-sample *t*-tests. The lower section reports disease duration and average daily alcohol use for the whole patient sample, as well as separately for males and females, along with the results of gender comparisons based on two-sample *t*-tests. DF, degrees of freedom; UA, Units of Alcohol.

### 2.2 Neurocognitive evaluation

AUD patients underwent the neuropsychological assessment before PET sessions. As previously reported (Galandra et al., 2018), both AUD patients and controls underwent the Brief Neuropsychological Examination (ENB-2, Mondini et al., 2011), a well-validated Italian battery for neuro-cognitive assessment that, in approximately 45 min, allows to assess different cognitive areas: attention (Trail Making Test A and B), memory (digit span, immediate and delayed prose memory), working-memory (interference memory tasks with 10- and 30-s delays), executive functions (TMT-B, cognitive estimation, abstract reasoning, phonemic fluency, clock drawing, overlapping figures), as well as perceptual and praxis skills (praxis abilities, spontaneous and copied drawing tasks, ideative and ideomotor praxis tests). The battery results in 15 scores of cognitive performance across these domains, alongside a score of global cognitive status.

### 2.3 Statistical analysis of neurocognitive data

Age and group effects were examined using parametric or non-parametric tests depending on a preliminary assessment of the normality of score distribution across all tasks. In case of significant effects of age or education level, we used an analysis of covariance (ANCOVA) to evaluate group differences in cognitive performance after accounting for these variables (Ciricugno et al., 2021). We applied a primary statistical threshold of *p* < 0.05 (two-tailed) corrected for multiple comparisons with False discovery Rate (FDR; Benjamini, 2010). We then explored higher-order cognitive domains transcending specific tasks through a Principal Component Analysis (PCA) on the 15 ENB-2 raw scores. The suitability of the correlation matrix was confirmed (Keiser-Meyer-Olkin Measure of Sampling Adequacy = 0.61; Bartlett’s test of sphericity<0.001; Supplementary Table 1). The number of components was determined using the Kaiser-Guttman criterion (eigenvalues>1), and an orthogonal rotation (Varimax) facilitated component interpretation (Supplementary Tables 2, 3). Finally, we performed an ANOVA (with FDR correction) on the PCA factor scores to investigate group differences in cognitive performance.

### 2.4 Acquisition and reconstruction of ^18^F-FDG PET image

In line with previous studies (e.g., Ritz et al., 2019) AUD patients underwent a ^18^F-FDG PET/CT scan acquisition (GE Discovery 690 VCT scanner) at rest, with their eyes closed, in a quiet and dark environment. We collected static emission images 45 min after injecting 2.5 MBq/Kg of ^18^F-FDG in fasting patients. This uptake time enables an equal distribution of the tracer across the entire brain, with negligible blood flow-dependent differences, and therefore an optimal signal-to-noise ratio (Della Rosa et al., 2014). PET data were acquired for 15 min, and images were reconstructed through a fully 3D ordered subset expectation maximization (3D-OSEM) algorithm. PET data correction (dead time, random, scatter, attenuation, normalization, TOF and point spread function) were included into the iterative reconstruction algorithm. A quality control process was performed to check for major artefacts in PET images due to inaccuracy in patient orientation or in PET-CT co-registration.

A General Electrics 3 Tesla MRI scanner (MR750 Discovery, GE Healthcare, Milwaukee, WI), equipped with a 16-channels head coil, was used to collect also a T1-weighted structural image through a 3D inversion-recovery-prepared fast spoiled gradient recalled (IR-FSPGR-BRAVO) sequence (152 contiguous slices, TR = 8.2 ms, TE = 3.2 ms, TI = 450 ms, in-plane resolution = 0.9375×0.9375 mm; thickness = 1 mm).

### 2.5 Spatial pre-processing of ^18^F-FDG PET images

We used the Statistical Parametrical Mapping (SPM12) software,^1^ as implemented in MATLAB (Mathworks Inc., Sherborn, MA, USA), to perform a standard pre-processing of PET scans. Each image was first normalized to an ^18^F-FDG PET template registered to the Montreal Neurological Institute (MNI) standard space (Della Rosa et al., 2014) using the default SPM12 bounding-box, resampled at an isotropic voxel size of 2 mm, and spatially smoothed with an 8 mm isotropic 3D Gaussian Full-Width-Hald-Maximum (FWHM) kernel. The ^18^F-FDG PET template has been reported to ensure high normalization accuracy, while reducing noise-related random effects (Della Rosa et al., 2014; Gallivanone et al., 2014). Each image was proportionally scaled to its global mean (Dukart et al., 2010; Signorini et al., 1999) to generate standardized uptake value ratio (SUVR) images and thus overcome issues of between-subject uptake variability (Buchert et al., 2005). We preferred this approach over other available scaling methods (e.g., cerebellar reference region) both because the former has been reported to enable higher signal-to-noise ratio (Nudo, 2013), and to ensure that intensity values would not be referenced to a single region underlying the executive processes under investigation such as cerebellum (Keren-Happuch et al., 2012). The same pre-processing pipeline was applied to 42 PET scans of age-matched healthy controls (henceforth “PET-HCs”), extracted from a larger, previously validated (Della Rosa et al., 2014), dataset of FDG-PET of healthy individuals.

### 2.6 Control analysis: correction for partial-volume effect

We performed a control analysis to assess partial volume effects (PVEs) in AUD patients’ FDG-PET brain images (Hoffman et al., 1979), using the modified Müller-Gärtner (mMG) approach as implemented in the PETPVE12 toolbox (Gonzalez-Escamilla et al., 2017). Each patient’s T1-weighted MRI image was co-registered to the corresponding FDG-PET image, and segmented into grey matter (GM), white matter (WM) and cerebrospinal fluid (CSF) using the CAT12^2^ toolbox. The mMG method uses the GM, WM and CSF compartments to correct the PET GM signal for spill-in effects from the surrounding tissue (typically WM signal) as well as spill-out effects of GM PET signal into adjacent WM and CSF compartments. In particular, the WM PET signal used to correct spill-in effects is estimated as signal spill-over between each pair of a pre-specified set of non-overlapping brain regions from the Desikan-Killiany (Desikan et al., 2006) structural atlas, as in the “Geometric Transfer Matrix” (GTM) method. The resulting PVE-corrected FDG images underwent the same pre-processing pipeline described (Section 2.5).

### 2.7 Statistical analyses of ^18^F-FDG PET images

SPM12 was also used for statistical analyses, including: (1) a two-sample t-test, to assess the hypothesis of decreased regional metabolism in AUD patients, compared with PET-HCs, while controlling for age (nuisance variable), and (2) multiple regressions, in the AUD sample, to assess a relationship between regional brain metabolism and performance in the cognitive domain(s) displaying a significant impairment in patients. In separate multiple regressions, age was modeled to either discount in correlation analyses, or to explicitly investigate in interaction analyses, its possible effect on the relationship between regional brain metabolism and cognitive performance. We used a conjunction analysis to assess the predicted anatomical overlap between the regions in which brain metabolism was lower in patients (vs. PET-HCs), and related to the extent of cognitive impairment. To assess the selectivity of results, we performed correlation and interaction control analyses with the remaining PCA scores.

For all contrasts the statistical threshold was set at *p* < 0.05, corrected for multiple comparisons with False discovery Rate (FDR; Chumbley and Friston, 2009; as in Arioli et al., 2021) at the cluster level (voxel-level forming threshold = *p* < 0.001 uncorrected). We additionally applied threshold-free cluster enhancement (TFCE; Smith and Nichols, 2009), that for each voxel in a cluster generates a TFCE score combining its statistical strength with cluster extent. By comparing the TFCE scores of the original data with a null distribution generated through 5,000 random permutations and correction for multiple comparisons, TFCE enables the identification of spatially extended signals without inflating false positives.

Based on behavioral (Section 3.1) and neural (Section 3.2) results, we assessed a moderation model to characterize the effect of age on the relationship between executive performance and brain metabolism in the right ventral anterior insula. To this purpose, we first used the SPM toolbox Marsbar^3^ to create binary masks of the right insular cluster displaying this interactive effect, and the toolbox Rex^4^ to extract its individual mean metabolism level. We then used the PROCESS macro (v.3.5) for SPSS (v.23, IBM, Armonk, NY, USA) to test Hayes’ (2017) model 1 (moderation), through a conditional process analysis (Hayes and Rockwood, 2020) based on Ordinary Least Squares (OLS) regression, using bootstrapping resampling (50,000 samples) to generate 95% confidence intervals for direct and moderated effects. Age and executive performance were mean centered before entering the analyses, and the Johnson and Neyman’s (1936) approach was used to compute the range of significance and simple slopes for the interaction analyses, which were assessed 1 SD below and above the mean. While a Breusch-Pagan test confirmed homoscedasticity of residuals (*p* = 0.25), due to a marginally significant negative correlation between metabolism and age (*r* = −0.3318, *p* = 0.1) we chose to use an heteroscedasticity-consistent standard error estimation (Hayes and Cai, 2007). The statistical threshold was set at *p* < 0.05 (two-tailed).

## 3 Results

### 3.1 Behavioral results

Age was negatively correlated with both recall scores (*r* = −0.31, *p* = 0.046) and TMT performance (positive correlation with response time; *r* = 0.46, *p* = 0.003), while no significant relationship with disease duration was found (*r* = 0.277, *p* = 0.201). Even when controlling the effect of age through an ANCOVA, AUD patients showed significantly worse performance than HCs in the ENB global score and in immediate recall, interference memory (10″), TMT-A, and overlapping figures (*p* < 0.05 FDR corrected; Supplementary Table 4).

A PCA allowed to reduce the 15 ENB-2 scores to 6 components explaining 74.89% of their variance (Supplementary Table 5) and covering cognitive domains such as visual-constructional abilities, verbal learning, basic-level and high-level executive processes, language and estimation-related processes (Supplementary Table 6). Significant group differences were found in the third component (basic-level executive processes) (*F*(1,39) = 11.58, *p* < 0.002) (Supplementary Table 7), primarily reflecting attention (strongest contribution from TMT-A response-time; *r* = −0.78, *p* < 0.001) and working-memory (strongest contribution from interference-memory-10″ (*r* = 0.71, *p* < 0.001) and 30″ (*r* = 0.69, *p* < 0.001)). Therefore, subsequent FDG-PET analyses focused on the relationship between brain metabolism and basic-level executive performance. The latter was negatively correlated with age (*r* = −0.4988, *p* = 0.018), while there was no significant correlation with disease duration (*r* = 0.1567, *p* = 0.486) or education level (*r* = 0.1372, *p* = 0.543). Accordingly, in subsequent PET analyses we investigated the effect of age on the relationship between executive performance and brain metabolism.

### 3.2 FDG-PET results

**Figure 1:**
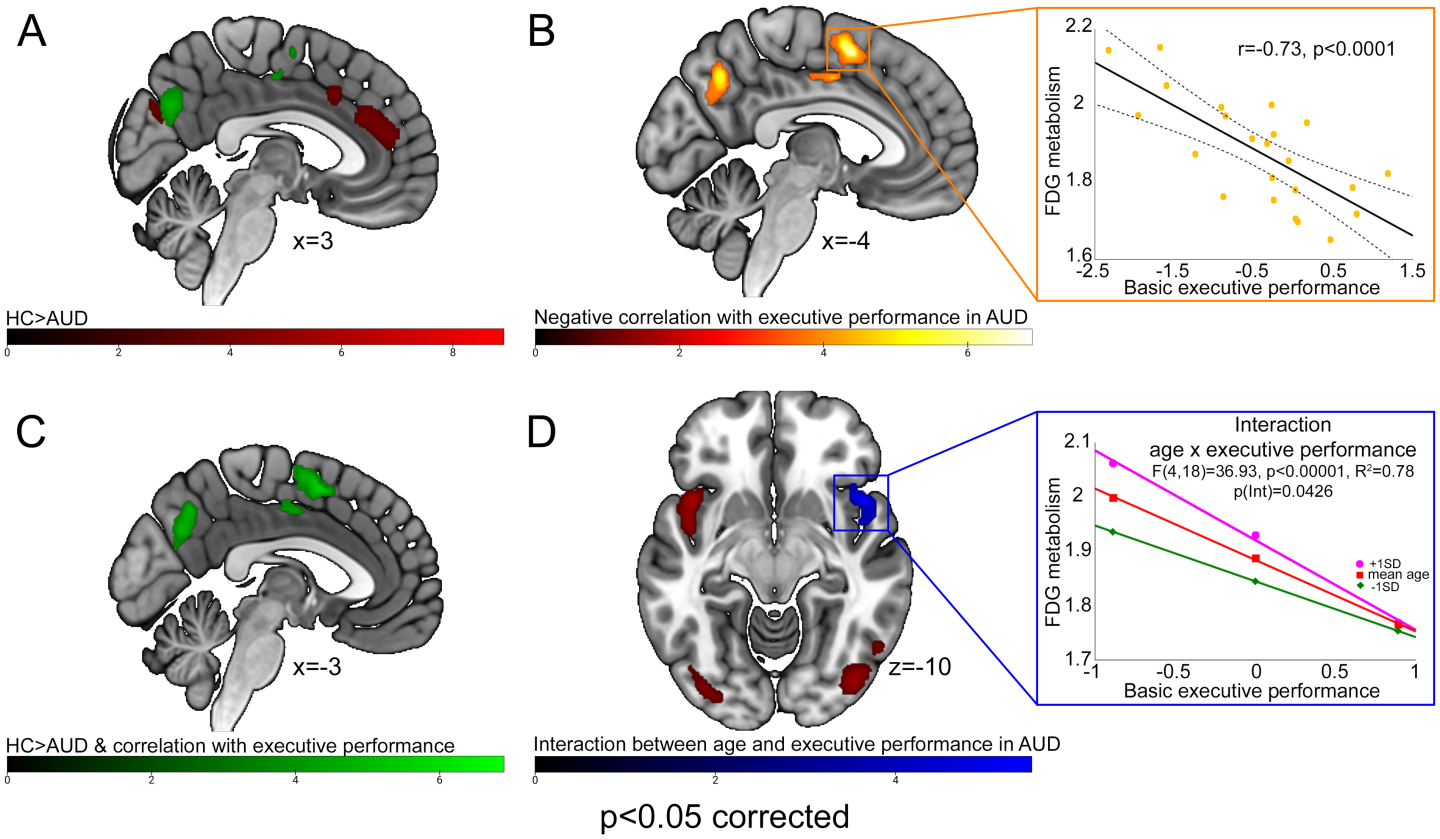
FDG-PET results. With different color-codes (as tracked by the respective colorbars), the figure depicts the regions in which brain metabolism was significantly lower in AUD patients compared with PET-HCs [red; **(A)**], negatively correlated with executive performance in the AUD sample [yellow; **(B)**], both significantly lower than PET-HCs, and negatively correlated with executive performance, in AUD patients [green; **(C)**], modulated by the interaction between age and executive performance [blue; **(D)**]. For AUD patients, scatterplots on the right depict the negative correlation between brain metabolism and executive performance alongside 95% confidence intervals (top), and its interaction with age as resulting from a conditional process analysis (bottom).

Brain metabolism was significantly lower in AUD patients, compared with PET-HCs, in the posterior frontomedial cortex (including the anterior and midcingulate cortex), left fronto-insular cortex (extending into the rolandic and parietal opercula) and right temporopolar and insular cortex. A bilateral hypometabolic pattern was found, in AUD patients, also in the posterior occipital cortex (inferior and middle occipital gyri), sensorimotor cortex and cerebellum (Figure 1A; Table 2a). Conversely, higher metabolism, in AUD patients than PET-HCs, was found in a sector of the thalamus projecting to the prefrontal cortex, and in the posterior cingulate cortex (Table 2b).

**Table 2:** FDG-PET correlates of executive impairment in AUD.

H	Brain region	Anatomy toolbox	x	y	z	T	K	*p*-corrected	TFCE
a) HC > AUD (hypometabolism in AUD)									
R	Anterior Cingulate Cortex		2	44	20	4.5	380	***p* = 0.0063**	**696.59**
	Middle Cingulate Cortex		2	18	38	4.14			
	Posterior-Medial Frontal		2	20	50	3.71			
L	Insula Lobe		−40	16	−2	6.23	1,556	***p* < 0.0001**	**1562.10**
	Precentral Gyrus	Area 44	−54	4	20	5.13			**1180.61**
	Insula lobe	Area OP4 [PV]	−56	−8	6	4.86			**1248.63**
	IFG (pars Opercularis)	Area 44	−52	10	20	4.86			**1171.62**
	Rolandic Operculum		−54	−2	6	4.74			**1245.72**
R	Temporal Pole/Insula lobe		36	14	−28	5.94	292	***p* = 0.0173**	**945.10**
L	Postcentral Gyrus	Area 3b	−44	−28	56	6.76	806	***p* < 0.001**	**1760.73**
	Inferior Parietal Lobule	Area 3b	−52	−20	40	5.68			**1242.19**
R	Postcentral Gyrus	Area 1	42	−36	64	6.47	647	***p* < 0.001**	**1353.39**
	Precentral Gyrus		50	−8	48	4.17			**938.81**
R	Middle Occipital Gyrus		30	−74	38	5.58	606	***p* < 0.001**	**1288.47**
	Cuneus		10	−78	32	5.43			**1288.84**
	Superior Occipital Gyrus		22	−80	22	3.76			**984.75**
L	Middle Occipital Gyrus	Area hOc4la	−42	−78	−2	4.54	453	***p* = 0.003**	**1133.04**
	Inferior Occipital Gyrus	Area hOc4la	−36	−84	−10	4.45			**1130.09**
	Middle Occipital Gyrus	Area PGp (IPL)	−40	−76	24	4.41			**1079.67**
	Fusiform Gyrus	Area FG2	−42	−72	−16	4.26			**1070.44**
R	Inferior Occipital Gyrus	Area hOc4v [V4(v)]	42	−78	−12	5.74	254	***p* = 0.0262**	**1208.16**
	Cerebellum	Lobule VIIa Crus1	−42	−46	−44	5.50	2,263	***p* < 0.0001**	
	Cerebellum	Lobule VIIa Crus1	56	−64	−34	5.26			
b) AUD > HC (hypermetabolism in AUD)									
R	Thalamus	Thal: prefrontal	12	−12	8	9.17	320	***p* < 0.0001**	**3522.22**
L	Thalamus	Thal: prefrontal	−16	−26	8	8.55	256	***p* < 0.001**	**3259.70**
L/R	Posterior cingulate cortex		10	−54	12	8.02	478	***p* < 0.0001**	**3646.82**
c) Negative correlation between brain metabolism and executive performance									
L	Posterior-Medial Frontal		−4	6	56	7.21	302	***p* = 0.0039**	**1292.20**
R	Middle cingulate cortex		8	−10	44	6.27			**1030.16**
L	Middle cingulate cortex		−4	−4	42	5.4			**984.59**
R	Posterior-Medial Frontal		6	−4	62	3.93			**950.02**
L	Medial precuneus		−4	−62	44	5.7	335	***p* = 0.0039**	**1150.01**
R	Medial precuneus		4	−66	32	4.95			**1103.26**
d) Common effects of a) and c)									
R	Middle cingulate cortex		8	−10	44	6.27	96	***p* < 0.001**	n/a
L	Middle cingulate cortex		−4	−4	42	5.4			n/a
L	Posterior-Medial Frontal		−4	6	56	7.21	182	***p* < 0.001**	n/a
R	Posterior-Medial Frontal		6	−4	62	3.93			n/a
L	Medial precuneus		−4	−62	44	5.7	302	***p* < 0.001**	n/a
R	Medial precuneus		4	−66	32	4.95			n/a
e) Age-related modulation of the relationship between brain metabolism and executive performance									
R	Anterior insula lobe		48	14	−10	5.68	235	***p* = 0.0135**	**809.64**

The table reports the regions in which brain metabolism was a) significantly lower in AUD patients compared with PET-HCs; b) significantly higher in AUD patients compared with PET-HCs; c) negatively correlated with executive performance in the AUD sample; d) both significantly lower than PET-HCs, and negatively correlated with executive performance, in AUD patients; e) modulated by the interaction between age and executive performance on regional brain metabolism.

AUD, alcoholic patients; L, left; H, hemisphere; OP, parietal operculum; IFG, inferior frontal gyrus; IPL, inferior parietal lobule; FG, fusiform gyrus; K, cluster extent in number of voxels (2 × 2 × 2 mm^3^); PET-HC, PET-healthy controls; R, right; TFCE, Threshold-Free-Cluster-Enhancement. Bold font indicates a statistically significant effect at *p* < 0.05, corrected for multiple comparisons at the cluster (K) or TFCE levels (consider that TFCE statistics are not available for conjunction analyses).

A negative correlation between basic executive performance and brain metabolism was found in the medial precuneus and in a posterior frontomedial cluster encompassing the pre-Supplementary Motor Area (pre-SMA) and the posterior dorsal midcingulate cortex (pdMCC), both located in the paracingulate gyrus according to the Harvard-Oxford structural atlas (Desikan et al., 2006; see also de la Vega et al., 2016) (Figure 1B; Table 2c). A conjunction analysis between the latter finding and the main effect of group (HC > AUD) highlighted, in AUD patients, an hypometabolic pattern inversely related to executive performance in the posterior frontomedial cortex and medial precuneus (Figures 1A,C; Table 2d).

An interaction analysis showed, in the right ventral anterior insula, a significant effect of age on the relationship between brain metabolism and executive performance (Figure 1D; Table 2e). Concerning the latter finding, a conditional process analysis highlighted a strongly significant model (*F*(4,18) = 36.9337, *p* < 0.00001; R^2^ = 0.7849) in which the right insular metabolism is not only inversely predicted by basic executive performance (*p* < 0.0001), but also by age (*p* = 0.0014) and their interactive effect (*p* = 0.0426) (Table 3). Namely, as shown in Figure 1D, age modulates this negative correlation only at the lowest level of executive performance (where right insular metabolism decreases with age), while such influence disappears at the highest executive skills.

**Table 3:** Interaction “age × executive performance” on right anterior insular metabolism.

Model summary						
R	R^2^	MSE	F	DF1	DF2	*p*
0.8859	0.785	0.004	36.935	4	18	**<0.0001**

Model						
	Coeff	SE	t	*p*	LLCI	ULCI
Executive performance	−0.125	0.012	−10.143	**<0.0001**	−0.151	−0.099
Age	−0.005	0.001	−3.786	**0.0014**	−0.008	−0.002
Interaction “Age × Executive performance”	0.003	0.001	2.182	**0.0426**	0.0001	0.006
Disease duration	0.041	0.028	1.452	0.1637	−0.018	0.101

Test(s) of highest order unconditional interaction(s)						
	R^2^-change	F	DF1	DF2	*p*	
Interaction “Age × Executive performance”	0.03	4.761	1	18	**0.0426**	

Conditional effects of posterior frontomedial metabolism at values of gender						
Moderator “Age”	Effect	SE	t	*p*	LLCI	ULCI
1 SD below mean	−0.148	0.016	−9.433	**<0.0001**	−0.1812	−0.115
Mean	−0.125	0.012	−10.143	**<0.0001**	−0.1514	−0.099
1 SD above mean	−0.103	0.017	−6.170	**<0.0001**	−0.1376	−0.068

Bootstrap results for regression model parameters					
	Coeff	BootMean	BootSe	BootLLCI	BootULCI
Executive performance	−0.125	−0.130	0.019	**−0.170**	**−0.095**
Age	−0.005	−0.005	0.002	**−0.009**	**−0.0011**
Interaction “Age × Executive performance”	0.003	0.004	0.003	**−0.0009**	**0.009**
Disease duration	0.041	0.043	0.035	−0.023	0.112

The table reports the coefficients and 95% confidence intervals of moderation for predicting right anterior insular metabolism based on AUD patients’ age and executive performance.

MSE, Mean Squared Error; DF, degrees of freedom; LLCI, lower level of confidence interval; ULCI, upper level of confidence interval; Coeff, coefficient; SE, standard error. Bold font denotes *p* < 0.05.

The lack of significant correlations or interactions with any of the other 5 PCA scores (all *p* > 0.05) confirmed the exclusivity of these findings to executive performance. Moreover, a whole-brain control analysis of PVE-corrected images (2.6) confirmed the statistical significance of both the negative correlation between executive performance and metabolism in the pdMCC (xyz = −2 –2 56; cluster-extent *p*-corrected = 0.024), and its interaction with age in the right anterior insula (xyz = 40 16 –6; cluster-extent *p*-corrected = 0.009) (Supplementary Figure 1). A homogeneity-of-slopes model highlighted a significant main effect of the “pre-processing” (uncorrected vs. PVE-corrected PET images) on pdMCC metabolism (*F*(1) = 28.65, *p* < 0.0001), yet with no interaction on the relationship with executive performance (F(1) = 1.02, *p* = 0.318) (see Supplementary Figure 1).

## 4 Discussion

We used ^18^F-FDG-PET to investigate the neural bases of cognitive-executive impairment in individuals with AUD. By focusing on a direct measure of brain metabolism, we complemented previous structural (e.g., Galandra et al., 2021; Li et al., 2021) and rs-*f*MRI (Galandra et al., 2019; Canessa et al., 2022; Suk et al., 2021) evidence that—in AUD—cognitive impairments might reflect an altered interplay between salience and default-mode networks.

Compared with PET-HCs, AUD patients displayed a widespread hypometabolic pattern including the anterior and midcingulate cortex, left fronto-insular cortex, right insular and temporopolar cortex, alongside the medial precuneus. In keeping with our previous structural (Galandra et al., 2018) and rs-*f*MRI (Canessa et al., 2022) evidence, these findings are suggestive of a functional impairment, in AUD, in the dACC and fronto-insular nodes of the salience network (Cushnie et al., 2023; Menon and Uddin, 2010; Seeley et al., 2007; Sridharan et al., 2008; Uddin, 2015). Altered synchronization has been reported, in AUD patients, between the insula and both the dorsolateral prefrontal and anterior cingulate nodes of the executive control and salience networks, respectively, possibly underlying an impaired access to executive resources required for regulating controlled behavior (Sullivan et al., 2013). This functional alteration is expected to impair the ability to detect and respond to salient stimuli, and, alongside the hypometabolic pattern in a key hub of the DMN such as the medial precuneus (Raichle, 2015; Sanz-Morales and Melero, 2024), might underpin an altered transition from automatic to controlled processing (Stavro et al., 2013; Yang et al., 2016). This dysfunctional mechanism could explain AUD patients’ difficulties in executive tasks requiring vigilance, sustained attention, attentional switching and working-memory (Chanraud et al., 2007; Jang et al., 2007; Sullivan et al., 2021; Trick et al., 2014; Wiers et al., 2015). A similar pattern—particularly involving the middle cingulate cortex (Pitel et al., 2009)—has been also reported in patients with KS (Maillard et al., 2021).

Importantly, however, we additionally found evidence of possible compensatory neural mechanisms in the same neural circuitry. Within the AUD sample, indeed, executive performance was inversely related to brain metabolism both in the medial precuneus and in the midcingulate cortex, that is considered to underpin attentional allocation and goal attainment as crucial drivers of cognitive control (Touroutoglou et al., 2020). In keeping with previous similar interpretations (Galandra et al., 2019; Sjoerds et al., 2017), the inverse efficiency-metabolism relationship suggests a compensatory neural mechanism, whereby higher activation in these areas at lower executive skills may help compensate for the functional outcome of the hypometabolic pattern discussed above. Alongside the observed hypermetabolism in a thalamic sector projecting to the prefrontal cortex (Shokri-Kojori et al., 2017), the heightened activity of these regions might thus represent an attempt to maintain cognitive efficiency despite an impaired transition between rest and effortful cognitive control due to altered functional and structural connections between their respective neural correlates in the default-mode and executive control networks (Crespi et al., 2020).

Our findings additionally suggest that this compensatory mechanism might also engage the right anterior insular sector of the salience network (Cai et al., 2014; Ghahremani et al., 2015; Molnar-Szakacs and Uddin, 2022), in which the negative relationship between brain metabolism and executive performance was also modulated by patients’ age. This modulation was only observed at the lowest level of executive performance, with right insular metabolism decreasing at increasing age, and disappeared at the highest executive skills (Figure 1D). This finding might reflect the previously reported hemispheric lateralization of insula connectivity (Kann et al., 2016), supporting a prominent role of the *right* anterior insula in salience processing (Ham et al., 2013), interplay with DMN (Bonnelle et al., 2012; Sridharan et al., 2008), and attentional orientation to salient stimuli (Thiebaut de Schotten et al., 2011). Along with behavioral evidence of decreased executive performance in older AUD patients, and of an age-related modulation of right insular activity (Kann et al., 2016), this finding might be suggestive of an age-related decline in the compensatory capacity of the right insular node of the salience network, further exacerbating executive impairments in older AUD patients. This hypothesis fits with previously reported neuroimaging evidence of age-related decreases in functional and structural brain integrity in AUD, particularly in regions involved in higher-order cognitive processes (Sullivan and Pfefferbaum, 2023).

Importantly, there is previous evidence of a relationship between altered insular metabolism (as tracked by regional cerebral blood flow; rCBF) and executive performance in AUD, that however specifically involves working-memory (Sullivan et al., 2021). While this finding fits with the present evidence of interference-memory as a key proxy of neuro-cognitive impairment in AUD, the multiple differences across studies—in terms of neuroimaging technique, neuropsychological battery, and sample characteristics—highlight the need of further studies addressing the insula role in AUD patients’ cognitive and behavioral changes. Moreover, while the observed hypometabolism in Crus I fits with previous related evidence of a cerebellum involvement in AUD (Ritz et al., 2019), the lack of hypermetabolism in cerebellar lobule VIII highlights inconsistencies that call for further investigation on this topic (see Arioli et al. (2022) for a similar discussion on Parkinson’s disease).

Overall, the observed interaction among brain metabolism, executive performance and age highlights the dynamic interplay of multiple factors in shaping cognitive efficiency in AUD, with the decreased effectiveness of compensatory mechanisms possibly explaining the larger executive impairment in older patients (Kuhns et al., 2022). Moreover, these findings support the view of cognitive decline in AUD as reflecting a specific pattern of network-level neural disruption involving multiple regions beyond the frontal cortex (Guggenmos et al., 2017).

There are some limitations to these findings. The relatively small sample-size limits the statistical power and generalizability of our results (particularly those concerning age effects on the relationship between executive performance and right insular metabolism) which should be therefore validated by future studies with larger cohorts. Moreover, its cross-sectional study design prevents the present study from drawing causal inferences about the relationship between brain metabolism and executive performance, which would rather require longitudinal studies enabling an explicit modeling of its temporal dynamics. A further limitation is represented by the lack of in-depth measures of executive functioning and cognitive control, such as response inhibition, that should be thoroughly assessed in future research to provide a more comprehensive understanding of executive dysfunction in AUD (Cao et al., 2023). In the trade-off between comprehensiveness and specificity, however, opting for a broad neuropsychological assessment allowed us to highlight the selective impairment of an executive domain transcending specific attentional and working-memory tasks. Finally, although patients joined the study after a 28-day alcohol withdrawal treatment and at least 10 days of detoxification, the present metabolic and cognitive data might be influenced by clinical factors known to be associated with AUD, such as thiamine deficiency (Davis and Bajaj, 2018; Dhir et al., 2019), hepatic encephalopathy (Davis and Bajaj, 2018) and benzodiazepine withdrawal (Podhorna, 2002) effects.

Notwithstanding these limitations and the need for follow-up studies, our findings have also important and multifaceted implications. First, they strengthen previous proposals about the role played by a dysfunctional salience network in AUD patients’ cognitive-executive impairment (Canessa et al., 2022; Suk et al., 2021; Zhu et al., 2017). Moreover, they highlight the potential of FDG-PET to complement structural and functional MRI data, thereby providing novel insights into the neural correlates of cognitive impairment in AUD. The unique information provided by FGD-PET could enhance the quality of differential diagnosis, particularly to distinguish among different neuropathological processes underlying cognitive decline in older patients. This consideration raises other important implications of the present findings, concerning the possible development of personalized treatment protocols. The observed age-related modulation of the relationship between brain metabolism and executive performance suggests that age should be considered in interventions aimed at enhancing cognitive functioning in AUD patients. For instance, younger patients may benefit more from strategies aimed at boosting compensatory mechanisms, such as cognitive training (Lo Presti et al., 2023), whereas older ones might require interventions directly addressing the underlying age-related hypometabolism. The latter approach may involve innovative treatment protocols such as neurostimulation, targeting specific nodes within the salience and/or executive networks (Mattavelli et al., 2022; Padula et al., 2022) to enhance cognitive control and reduce relapse rates (Dubuson et al., 2021; Herremans et al., 2015; Mishra et al., 2010).

In conclusion, through FDG-PET we showed that the well-known executive impairment in AUD, mainly involving attention and working-memory (Galandra et al., 2018), reflects a decrease of brain metabolism in key nodes of the salience and cognitive control networks, that at the lowest level of cognitive efficiency is steeper in in older patients. These findings highlight the potential of FDG-PET as a valuable tool for understanding the functional underpinnings of cognitive decline in AUD, and for supporting the design of personalized interventions.

## Data Availability

The datasets presented in this article are not readily available because the data that support the findings of this study are available from the corresponding author upon reasonable request. Requests to access the datasets should be directed to nicola.canessa@iusspavia.it.

## Footnotes

^1^ https://www.fil.ion.ucl.ac.uk/spm/software/spm12/

^2^ www.neuro.uni-jena.de/cat/

^3^ http://marsbar.sourceforge.net/

^4^ https://www.nitrc.org/projects/rex/

## References

[ref1] Alarcon R. Nalpas B. Pelletier S. Perney P. (2015). Mo CA as a screening tool of neuropsychological deficits in alcohol-dependent patients. Alcohol. Clin. Exp. Res. 39, 1042–1048. doi: 10.1111/acer.12734, PMID: 25939560

[ref9001] Arioli M. Basso G. Carne I. Poggi P. Canessa N. (2021). Increased pSTS activity and decreased pSTS-mPFC connectivity when processing negative social interactions. Behav. Brain Res. 399:113027. doi: 10.1016/j.bbr.2020.113027 33249070

[ref2] Arioli M. Cattaneo Z. Rusconi M. L. Blandini F. Tettamanti M. (2022). Action and emotion perception in Parkinson’s disease: a neuroimaging meta-analysis. NeuroImage Clin. 35:103031. doi: 10.1016/j.nicl.2022.103031, PMID: 35569229 PMC9112018

[ref3] Benjamini Y. (2010). Discovering the false discovery rate. J. R. Stat. Soc. Ser. B Stat. Method. 72, 405–416. doi: 10.1111/j.1467-9868.2010.00746.x

[ref4] Bickel W. K. Moody L. N. Eddy C. R. Franck C. T. (2017). Neurocognitive dysfunction in addiction: testing hypotheses of diffuse versus selective phenotypic dysfunction with a classification-based approach. Exp. Clin. Psychopharmacol. 25, 322–332. doi: 10.1037/pha0000115, PMID: 28782983 PMC5606154

[ref5] Bonnelle V. Ham T. E. Leech R. Kinnunen K. M. Mehta M. A. Greenwood R. J.. (2012). Salience network integrity predicts default mode network function after traumatic brain injury. Proc. Natl. Acad. Sci. 109, 4690–4695. doi: 10.1073/pnas.1113455109, PMID: 22393019 PMC3311356

[ref6] Bralet M. C. Mitelman S. A. Goodman C. R. Lincoln S. Hazlett E. A. Buchsbaum M. S. (2022). Fluorodeoxyglucose positron emission tomography scans in patients with alcohol use disorder. Alcohol. Clin. Exp. Res. 46, 994–1010. doi: 10.1111/acer.14845, PMID: 35451074

[ref7] Buchert R. Wilke F. Chakrabarti B. Martin B. Brenner W. Mester J.. (2005). Adjusted scaling of FDG positron emission tomography images for statistical evaluation in patients with suspected Alzheimer’s disease. J. Neuroimaging 15, 348–355. doi: 10.1111/j.1552-6569.2005.tb00335.x, PMID: 16254400

[ref9003] Butcher T. J. Chumin E. J. West J. D. Dzemidzic M. Yoder K. K. (2022). Cerebral blood flow in the salience network of individuals with alcohol use disorder. Alcohol and Alcoholism 57, 445–451. doi: 10.1093/alcalc/agab062 34541599 PMC9613478

[ref8] Cai W. Ryali S. Chen T. Li C. S. R. Menon V. (2014). Dissociable roles of right inferior frontal cortex and anterior insula in inhibitory control: evidence from intrinsic and task-related functional parcellation, connectivity, and response profile analyses across multiple datasets. J. Neurosci. 34, 14652–14667. doi: 10.1523/JNEUROSCI.3048-14.2014, PMID: 25355218 PMC4212065

[ref9] Camchong J. Stenger A. Fein G. (2013). Resting-state synchrony in long-term abstinent alcoholics. Alcohol. Clin. Exp. Res. 37, 75–85. doi: 10.1111/j.1530-0277.2012.01859.x, PMID: 22725701 PMC3493852

[ref10] Canessa N. Basso G. Manera M. Poggi P. Gianelli C. (2022). Functional coherence in intrinsic frontal executive networks predicts cognitive impairments in alcohol use disorder. Brain Sci. 13:45. doi: 10.3390/brainsci13010045, PMID: 36672027 PMC9856140

[ref11] Cao Y. Tian F. Zeng J. Gong Q. Yang X. Jia Z. (2023). The brain activity pattern in alcohol-use disorders under inhibition response task. J. Psychiatr. Res. 163, 127–134. doi: 10.1016/j.jpsychires.2023.05.009, PMID: 37209618

[ref12] Chanraud S. Martelli C. Delain F. Kostogianni N. Douaud G. Aubin H. J.. (2007). Brain morphometry and cognitive performance in detoxified alcohol-dependents with preserved psychosocial functioning. Neuropsychopharmacology 32, 429–438. doi: 10.1038/sj.npp.1301219, PMID: 17047671

[ref13] Chanraud S. Pitel A. L. Müller-Oehring E. M. Pfefferbaum A. Sullivan E. V. (2013). Remapping the brain to compensate for impairment in recovering alcoholics. Cereb. Cortex 23, 97–104. doi: 10.1093/cercor/bhr381, PMID: 22275479 PMC3513953

[ref14] Chanraud S. Pitel A. L. Pfefferbaum A. Sullivan E. V. (2011). Disruption of functional connectivity of the default-mode network in alcoholism. Cereb. Cortex 21, 2272–2281. doi: 10.1093/cercor/bhq297, PMID: 21368086 PMC3169657

[ref15] Chumbley J. R. Friston K. J. (2009). False discovery rate revisited: FDR and topological inference using Gaussian random fields. NeuroImage 44, 62–70. doi: 10.1016/j.neuroimage.2008.05.021, PMID: 18603449

[ref16] Ciricugno A. Bartlett M. L. Gwinn O. S. Carragher D. J. Nicholls M. E. (2021). The effect of cognitive load on horizontal and vertical spatial asymmetries. Laterality 26, 706–724. doi: 10.1080/1357650X.2021.1920972, PMID: 33906579

[ref9002] Clergue-Duval V. Questel F. Azuar J. Paquet C. Cognat E. Amami J.. (2020). Brain 18FDG-PET pattern in patients with alcohol-related cognitive impairment. European J. Nuclear Med. Mol. Imaging 47, 281–291. doi: 10.1007/s00259-019-04487-1 31428832

[ref17] Cotta Ramusino M. Massa F. Festari C. Gandolfo F. Nicolosi V. Orini S.. (2024). Diagnostic performance of molecular imaging methods in predicting the progression from mild cognitive impairment to dementia: an updated systematic review. Eur. J. Nucl. Med. Mol. Imaging 51, 1876–1890. doi: 10.1007/s00259-024-06631-y 38355740

[ref18] Crespi C. Galandra C. Canessa N. Manera M. Poggi P. Basso G. (2020). Microstructural damage of white-matter tracts connecting large-scale networks is related to impaired executive profile in alcohol use disorder. NeuroImage Clin. 25:102141. doi: 10.1016/j.nicl.2019.102141, PMID: 31927501 PMC6953958

[ref19] Crespi C. Galandra C. Manera M. Basso G. Poggi P. Canessa N. (2019). Executive impairment in alcohol use disorder reflects structural changes in large-scale brain networks: a joint independent component analysis on gray-matter and white-matter features. Front. Psychol. 10:2479. doi: 10.3389/fpsyg.2019.02479, PMID: 32038340 PMC6988803

[ref20] Cushnie A. K. Tang W. Heilbronner S. R. (2023). Connecting circuits with networks in addiction neuroscience: a salience network perspective. Int. J. Mol. Sci. 24:9083. doi: 10.3390/ijms24109083, PMID: 37240428 PMC10219092

[ref21] Dao-Castellana M. H. Samson Y. Legault F. Martinot J. L. Aubin H. J. Crouzel C.. (1998). Frontal dysfunction in neurologically normal chronic alcoholic subjects: metabolic and neuropsychological findings. Psychol. Med. 28, 1039–1048. doi: 10.1017/S0033291798006849, PMID: 9794011

[ref9004] Davis B. C. Bajaj J. S. (2018). Effects of alcohol on the brain in cirrhosis: beyond hepatic encephalopathy. Alcoholism: Clin. Experimental Res. 42, 660–667. doi: 10.1111/acer.13605 29417604

[ref9005] de la Vega A. Chang L. J. Banich M. T. Wager T. D. Yarkoni T. (2016). Large-scale meta-analysis of human medial frontal cortex reveals tripartite functional organization. J. Neurosci. 36, 6553–6562. doi: 10.1523/JNEUROSCI.4402-15.2016 27307242 PMC5015787

[ref22] Della Rosa P. A. Cerami C. Gallivanone F. Prestia A. Caroli A. Castiglioni I.. (2014). A standardized [18F]-FDG-PET template for spatial normalization in statistical parametric mapping of dementia. Neuroinformatics 12, 575–593. doi: 10.1007/s12021-014-9235-4, PMID: 24952892

[ref23] Desikan R. S. Ségonne F. Fischl B. Quinn B. T. Dickerson B. C. Blacker D.. (2006). An automated labeling system for subdividing the human cerebral cortex on MRI scans into gyral based regions of interest. NeuroImage 31, 968–980. doi: 10.1016/j.neuroimage.2006.01.021, PMID: 16530430

[ref9006] Dhir S. Tarasenko M. Napoli E. Giulivi C. (2019). Neurological, psychiatric, and biochemical aspects of thiamine deficiency in children and adults. Front. Psychiatry 10:447129. doi: 10.3389/fpsyt.2019.00207 PMC6459027 31019473

[ref24] Dubuson M. Kornreich C. Vanderhasselt M. A. Baeken C. Wyckmans F. Dousset C.. (2021). Transcranial direct current stimulation combined with alcohol cue inhibitory control training reduces the risk of early alcohol relapse: a randomized placebo-controlled clinical trial. Brain Stimul. 14, 1531–1543. doi: 10.1016/j.brs.2021.10.386, PMID: 34687964

[ref25] Dukart J. Mueller K. Horstmann A. Vogt B. Frisch S. Barthel H.. (2010). Differential effects of global and cerebellar normalization on detection and differentiation of dementia in FDG-PET studies. NeuroImage 49, 1490–1495. doi: 10.1016/j.neuroimage.2009.09.017, PMID: 19770055

[ref26] Ewers M. Brendel M. Rizk-Jackson A. Rominger A. Bartenstein P. Schuff N.. (2014). Reduced FDG-PET brain metabolism and executive function predict clinical progression in elderly healthy subjects. NeuroImage Clin. 4, 45–52. doi: 10.1016/j.nicl.2013.10.018, PMID: 24286024 PMC3841292

[ref27] Fama R. Le Berre A. P. Sassoon S. A. Zahr N. M. Pohl K. M. Pfefferbaum A.. (2019). Relations between cognitive and motor deficits and regional brain volumes in individuals with alcoholism. Brain Struct. Funct. 224, 2087–2101. doi: 10.1007/s00429-019-01894-w, PMID: 31161472 PMC7082221

[ref28] Fede S. J. Grodin E. N. Dean S. F. Diazgranados N. Momenan R. (2019). Resting state connectivity best predicts alcohol use severity in moderate to heavy alcohol users. NeuroImage Clin. 22:101782. doi: 10.1016/j.nicl.2019.101782, PMID: 30921611 PMC6438989

[ref29] Fox M. D. Raichle M. E. (2007). Spontaneous fluctuations in brain activity observed with functional magnetic resonance imaging. Nat. Rev. Neurosci. 8, 700–711. doi: 10.1038/nrn2201, PMID: 17704812

[ref30] Galandra C. Basso G. Manera M. Crespi C. Giorgi I. Vittadini G.. (2018). Salience network structural integrity predicts executive impairment in alcohol use disorders. Sci. Rep. 8:14481. doi: 10.1038/s41598-018-32828-x, PMID: 30262893 PMC6160480

[ref31] Galandra C. Basso G. Manera M. Crespi C. Giorgi I. Vittadini G.. (2019). Abnormal fronto-striatal intrinsic connectivity reflects executive dysfunction in alcohol use disorders. Cortex 115, 27–42. doi: 10.1016/j.cortex.2019.01.004, PMID: 30738999

[ref9009] Galandra C. Crespi C. Basso G. Manera M. R. Giorgi I. Poggi P.. (2021). Decreased information processing speed and decision-making performance in alcohol use disorder: combined neurostructural evidence from VBM and TBSS. Brain Imaging Behav. 15, 205–215. doi: 10.1007/s11682-019-00248-8 32124275

[ref32] Gallivanone F. Della Rosa P. A. Perani D. Gilardi M. C. Castiglioni I. (2014). The impact of different 18FDG PET healthy subject scans for comparison with single patient in SPM analysis. Q. J. Nucl. Med. Mol. Imaging 61, 115–132. doi: 10.23736/S1824-4785.16.02749-7 25479418

[ref33] Ghahremani A. Rastogi A. Lam S. (2015). The role of right anterior insula and salience processing in inhibitory control. J. Neurosci. 35, 3291–3292. doi: 10.1523/JNEUROSCI.5239-14.2015, PMID: 25716829 PMC6605563

[ref34] Gonzalez-Escamilla G. Lange C. Teipel S. Buchert R. Grothe M. J. Alzheimer’s Disease Neuroimaging Initiative (2017). PETPVE12: an SPM toolbox for partial volume effects correction in brain PET–application to amyloid imaging with AV45-PET. NeuroImage 147, 669–677. doi: 10.1016/j.neuroimage.2016.12.077, PMID: 28039094

[ref9010] Goulden N. Khusnulina A. Davis N. J. Bracewell R. M. Bokde A. L. McNulty J. P.. (2014). The salience network is responsible for switching between the default mode network and the central executive network: replication from DCM. Neuroimage 99, 180–190. doi: 10.1016/j.neuroimage.2014.05.052 24862074

[ref35] Green A. Garrick T. Sheedy D. Blake H. Shores E. A. Harper C. (2010). The effect of moderate to heavy alcohol consumption on neuropsychological performance as measured by the repeatable battery for the assessment of neuropsychological status. Alcohol. Clin. Exp. Res. 34, 443–450. doi: 10.1111/j.1530-0277.2009.01108.x, PMID: 20028356

[ref36] Grodin E. N. Cortes C. R. Spagnolo P. A. Momenan R. (2017). Structural deficits in salience network regions are associated with increased impulsivity and compulsivity in alcohol dependence. Drug Alcohol Depend. 179, 100–108. doi: 10.1016/j.drugalcdep.2017.06.014, PMID: 28763777 PMC11034794

[ref37] Guggenmos M. Schmack K. Sekutowicz M. Garbusow M. Sebold M. Sommer C.. (2017). Quantitative neurobiological evidence for accelerated brain aging in alcohol dependence. Transl. Psychiatry 7:1279. doi: 10.1038/s41398-017-0037-y, PMID: 29225356 PMC5802586

[ref38] Guillén E. F. Rosales J. J. Lisei D. Grisanti F. Riverol M. Arbizu J. (2020). Current role of 18F-FDG-PET in the differential diagnosis of the main forms of dementia. Clin. Translat. Imaging 8, 127–140. doi: 10.1007/s40336-020-00366-0

[ref39] Hahn S. Mackey S. Cousijn J. Foxe J. J. Heinz A. Hester R.. (2022). Predicting alcohol dependence from multi-site brain structural measures. Hum. Brain Mapp. 43, 555–565. doi: 10.1002/hbm.25248, PMID: 33064342 PMC8675424

[ref40] Ham T. Leff A. de Boissezon X. Joffe A. Sharp D. J. (2013). Cognitive control and the salience network: an investigation of error processing and effective connectivity. J. Neurosci. 33, 7091–7098. doi: 10.1523/JNEUROSCI.4692-12.2013, PMID: 23595766 PMC6618896

[ref41] Hayes A. F. (2017). Introduction to mediation, moderation, and conditional process analysis: A regression-based approach. New York City: Guilford Publications.

[ref42] Hayes A. F. Cai L. (2007). Using heteroskedasticity-consistent standard error estimators in OLS regression: an introduction and software implementation. Behav. Res. Methods 39, 709–722. doi: 10.3758/BF03192961, PMID: 18183883

[ref43] Hayes A. F. Rockwood N. J. (2020). Conditional process analysis: concepts, computation, and advances in the modeling of the contingencies of mechanisms. Am. Behav. Sci. 64, 19–54. doi: 10.1177/0002764219859633

[ref44] Herremans S. C. Van Schuerbeek P. De Raedt R. Matthys F. Buyl R. De Mey J.. (2015). The impact of accelerated right prefrontal high-frequency repetitive transcranial magnetic stimulation (rTMS) on cue-reactivity: an fMRI study on craving in recently detoxified alcohol-dependent patients. PLoS One 10:e0136182. doi: 10.1371/journal.pone.0136182, PMID: 26295336 PMC4546410

[ref45] Hoffman E. J. Huang S. C. Phelps M. E. (1979). Quantitation in positron emission computed tomography: 1. Effect of object size. J. Comput. Assist. Tomogr. 3, 299–308. doi: 10.1097/00004728-197906000-00001, PMID: 438372

[ref47] Jang D. P. Namkoong K. Kim J. J. Park S. Kim I. Y. Kim S. I.. (2007). The relationship between brain morphometry and neuropsychological performance in alcohol dependence. Neurosci. Lett. 428, 21–26. doi: 10.1016/j.neulet.2007.09.047, PMID: 17951002

[ref48] Johnson P. O. Neyman J. (1936). Tests of certain linear hypotheses and their application to some educational problems. Stat. Res. Mem. 1, 57–63.

[ref9007] Kann S. Zhang S. Manza P. Leung H. C. Li C. S. R. (2016). Hemispheric lateralization of resting-state functional connectivity of the anterior insula: association with age, gender, and a novelty-seeking trait. Brain Connectivity 6, 724–734. doi: 10.1089/brain.2016.0443 27604154 PMC5105339

[ref49] Keren-Happuch E. Chen S. H. A. Ho M. H. R. Desmond J. E. (2012). A meta-analysis of cerebellar contributions to higher cognition from PET and fMRI studies. Hum. Brain Mapp. 35:593. doi: 10.1002/hbm.22194 23125108 PMC3866223

[ref50] Kuhns L. Kroon E. Lesscher H. Mies G. Cousijn J. (2022). Age-related differences in the effect of chronic alcohol on cognition and the brain: a systematic review. Transl. Psychiatry 12:345. doi: 10.1038/s41398-022-02100-y, PMID: 36008381 PMC9411553

[ref51] Li L. Yu H. Liu Y. Meng Y. J. Li X. J. Zhang C.. (2021). Lower regional grey matter in alcohol use disorders: evidence from a voxel-based meta-analysis. BMC Psychiatry 21:247. doi: 10.1186/s12888-021-03244-9, PMID: 33975595 PMC8111920

[ref52] Lo Presti S. Origlia S. Gianelli C. Canessa N. (2023). Cognition, body, and mind: a three-in-one coordinate-based fMRI meta-analysis on cognitive, physical, and meditative trainings. Hum. Brain Mapp. 44, 3795–3814. doi: 10.1002/hbm.26312, PMID: 37067079 PMC10203812

[ref53] Maharjan S. Amjad Z. Abaza A. Vasavada A. M. Sadhu A. Valencia C.. (2022). Executive dysfunction in patients with alcohol use disorder: a systematic review. Cureus 14:e29207. doi: 10.7759/cureus.29207, PMID: 36258974 PMC9573267

[ref9008] Maillard A. Laniepce A. Cabé N. Boudehent C. Chételat G. Urso L.. (2021). Temporal cognitive and brain changes in Korsakoff syndrome. Neurol. 96, e1987–e1998. doi: 10.1212/WNL.0000000000011749 33637634

[ref54] Maleki N. Sawyer K. S. Levy S. Harris G. J. Oscar-Berman M. (2022). Intrinsic brain functional connectivity patterns in alcohol use disorder. Brain Commun. 4:fcac290. doi: 10.1093/braincomms/fcac290, PMID: 36419966 PMC9679426

[ref55] Mattavelli G. Lo Presti S. Tornaghi D. Canessa N. (2022). High-definition transcranial direct current stimulation of the dorsal anterior cingulate cortex modulates decision-making and executive control. Brain Struct. Funct. 227, 1565–1576. doi: 10.1007/s00429-022-02456-3, PMID: 35102442

[ref56] Menon V. Uddin L. Q. (2010). Saliency, switching, attention and control: a network model of insula function. Brain Struct. Funct. 214, 655–667. doi: 10.1007/s00429-010-0262-0, PMID: 20512370 PMC2899886

[ref57] Mishra B. R. Nizamie S. H. Das B. Praharaj S. K. (2010). Efficacy of repetitive transcranial magnetic stimulation in alcohol dependence: a sham-controlled study. Addiction 105, 49–55. doi: 10.1111/j.1360-0443.2009.02777.x, PMID: 20078462

[ref58] Molnar-Szakacs I. Uddin L. Q. (2022). Anterior insula as a gatekeeper of executive control. Neurosci. Biobehav. Rev. 139:104736. doi: 10.1016/j.neubiorev.2022.104736, PMID: 35700753

[ref59] Mondini S. Mapelli D. Vestri A. Arcara G. Bisiacchi P. (2011). Esame neuropsicologico breve. Una batteria di test per lo screening neuropsicologico. Milan: Raffaello Cortina.

[ref60] Müller-Oehring E. M. Jung Y. C. Pfefferbaum A. Sullivan E. V. Schulte T. (2015). The resting brain of alcoholics. Cereb. Cortex 25, 4155–4168. doi: 10.1093/cercor/bhu134, PMID: 24935777 PMC4816777

[ref61] Müller-Oehring E. M. Jung Y. C. Sullivan E. V. Hawkes W. C. Pfefferbaum A. Schulte T. (2013). Midbrain-driven emotion and reward processing in alcoholism. Neuropsychopharmacology 38, 1844–1853. doi: 10.1038/npp.2013.102, PMID: 23615665 PMC3746685

[ref62] Nestor P. J. Altomare D. Festari C. Drzezga A. Rivolta J. Walker Z.. (2018). Clinical utility of FDG-PET for the differential diagnosis among the main forms of dementia. Eur. J. Nucl. Med. Mol. Imaging 45, 1509–1525. doi: 10.1007/s00259-018-4035-y, PMID: 29736698

[ref63] Nudo R. J. (2013). Recovery after brain injury: mechanisms and principles. Front. Hum. Neurosci. 7:887. doi: 10.3389/fnhum.2013.00887, PMID: 24399951 PMC3870954

[ref64] Oscar-Berman M. Marinkovic K. (2003). Alcoholism and the brain: an overview. Alcohol Res. Health 27, 125–133, PMID: 15303622 PMC6668884

[ref9011] Podhorna J. (2002). The experimental pharmacotherapy of benzodiazepine withdrawal. Curr. Pharmaceutical Design 8, 23–43. doi: 10.2174/1381612023396636 11812248

[ref65] Padula C. B. Tenekedjieva L. T. McCalley D. M. Al-Dasouqi H. Hanlon C. A. Williams L. M.. (2022). Targeting the salience network: a mini-review on a novel neuromodulation approach for treating alcohol use disorder. Front. Psych. 13:893833. doi: 10.3389/fpsyt.2022.893833, PMID: 35656355 PMC9152026

[ref66] Peters S. K. Dunlop K. Downar J. (2016). Cortico-striatal-thalamic loop circuits of the salience network: a central pathway in psychiatric disease and treatment. Front. Syst. Neurosci. 10:104. doi: 10.3389/fnsys.2016.00104, PMID: 28082874 PMC5187454

[ref67] Pitel A. L. Rivier J. Beaunieux H. Vabret F. Desgranges B. Eustache F. (2009). Changes in the episodic memory and executive functions of abstinent and relapsed alcoholics over a 6-month period. Alcohol. Clin. Exp. Res. 33, 490–498. doi: 10.1111/j.1530-0277.2008.00859.x, PMID: 19120052

[ref68] Raichle M. E. (2015). The brain’s default mode network. Annu. Rev. Neurosci. 38, 433–447. doi: 10.1146/annurev-neuro-071013-014030, PMID: 25938726

[ref69] Ramey T. Regier P. S. (2019). Cognitive impairment in substance use disorders. CNS Spectr. 24, 102–113. doi: 10.1017/S1092852918001426, PMID: 30591083 PMC6599555

[ref9013] Ritz L. Segobin S. Lannuzel C. Boudehent C. Vabret F. Eustache F.. (2016). Direct voxel-based comparisons between grey matter shrinkage and glucose hypometabolism in chronic alcoholism. J. Cerebral Blood Flow & Metabolism 36, 1625–1640. doi: 10.1177/0271678X15611136 PMC5012518 26661206

[ref9014] Ritz L. Segobin S. Lannuzel C. Laniepce A. Boudehent C. Cabé N.. (2019). Cerebellar hypermetabolism in alcohol use disorder: compensatory mechanism or maladaptive plasticity?. Alcoholism: Clinical and Experimental Res. 43, 2212–2221. doi: 10.1111/acer.14158 31373706

[ref70] Sanz-Morales E. Melero H. (2024). Advances in the fMRI analysis of the default mode network: a review. Brain Struct. Funct. 230:22. doi: 10.1007/s00429-024-02888-z, PMID: 39738718

[ref71] Seeley W. W. Menon V. Schatzberg A. F. Keller J. Glover G. H. Kenna H.. (2007). Dissociable intrinsic connectivity networks for salience processing and executive control. J. Neurosci. 27, 2349–2356. doi: 10.1523/JNEUROSCI.5587-06.2007, PMID: 17329432 PMC2680293

[ref9015] Segobin S. La Joie R. Ritz L. Beaunieux H. Desgranges B. Chételat G.. (2015). FDG-PET contributions to the pathophysiology of memory impairment. Neuropsychol. Rev. 25, 326–355. doi: 10.1007/s11065-015-9297-6 26319237

[ref72] Shokri-Kojori E. Tomasi D. Wiers C. E. Wang G.-J. Volkow N. D. (2017). Alcohol affects brain functional connectivity and its coupling with behavior: greater effects in male heavy drinkers. Mol. Psychiatry 22, 1185–1195. doi: 10.1038/mp.2016.25, PMID: 27021821 PMC5138152

[ref73] Signorini M. Paulesu E. Friston K. Perani D. Colleluori A. Lucignani G.. (1999). Rapid assessment of regional cerebral metabolic abnormalities in single subjects with quantitative and nonquantitative [18F] FDG PET: a clinical validation of statistical parametric mapping. NeuroImage 9, 63–80. doi: 10.1006/nimg.1998.0381, PMID: 9918728

[ref74] Sjoerds Z. Stufflebeam S. M. Veltman D. J. Van den Brink W. Penninx B. W. Douw L. (2017). Loss of brain graph network efficiency in alcohol dependence. Addict. Biol. 22, 523–534. doi: 10.1111/adb.12346, PMID: 26692359 PMC4917471

[ref75] Smith S. M. Nichols T. E. (2009). Threshold-free cluster enhancement: addressing problems of smoothing, threshold dependence and localisation in cluster inference. NeuroImage 44, 83–98. doi: 10.1016/j.neuroimage.2008.03.061, PMID: 18501637

[ref76] Song Z. Chen J. Wen Z. Zhang L. (2021). Abnormal functional connectivity and effective connectivity between the default mode network and attention networks in patients with alcohol-use disorder. Acta Radiol. 62, 251–259. doi: 10.1177/0284185120923270, PMID: 32423229

[ref77] Spindler C. Mallien L. Trautmann S. Alexander N. Muehlhan M. (2022). A coordinate-based meta-analysis of white matter alterations in patients with alcohol use disorder. Transl. Psychiatry 12:40. doi: 10.1038/s41398-022-01809-0, PMID: 35087021 PMC8795454

[ref78] Spitta G. Garbusow M. Buchert R. Heinz A. (2023). Dopamine and alcohol: a review of in vivo PET and SPECT studies. Neuropsychobiology 82, 319–345. doi: 10.1159/000534620, PMID: 37963449

[ref79] Sridharan D. Levitin D. J. Menon V. (2008). A critical role for the right fronto-insular cortex in switching between central-executive and default-mode networks. Proc. Natl. Acad. Sci. 105, 12569–12574. doi: 10.1073/pnas.0800005105, PMID: 18723676 PMC2527952

[ref80] Stavro K. Pelletier J. Potvin S. (2013). Widespread and sustained cognitive deficits in alcoholism: a meta-analysis. Addict. Biol. 18, 203–213. doi: 10.1111/j.1369-1600.2011.00418.x, PMID: 22264351

[ref81] Suk J. W. Hwang S. Cheong C. (2021). Functional and structural alteration of default mode, executive control, and salience networks in alcohol use disorder. Front. Psych. 12:742228. doi: 10.3389/fpsyt.2021.742228, PMID: 34744834 PMC8564495

[ref82] Sullivan E. V. Müller-Oehring E. Pitel A. L. Chanraud S. Shankaranarayanan A. Alsop D. C.. (2013). A selective insular perfusion deficit contributes to compromised salience network connectivity in recovering alcoholic men. Biol. Psychiatry 74, 547–555. doi: 10.1016/j.biopsych.2013.02.026, PMID: 23587427 PMC3766441

[ref83] Sullivan E. V. Pfefferbaum A. (2023). Alcohol use disorder: neuroimaging evidence for accelerated aging of brain morphology and hypothesized contribution to age-related dementia. Alcohol 107, 44–55. doi: 10.1016/j.alcohol.2022.06.002, PMID: 35781021 PMC11424507

[ref9012] Sullivan E. V. Zhao Q. Pohl K. M. Zahr N. M. Pfefferbaum A. (2021). Attenuated cerebral blood flow in frontolimbic and insular cortices in alcohol use disorder: relation to working memory. J. Psychiatric Res. 136, 140–148. doi: 10.1016/j.jpsychires.2021.01.053 PMC8009820 33592385

[ref84] Taebi A. Becker B. Klugah-Brown B. Roecher E. Biswal B. Zweerings J.. (2022). Shared network-level functional alterations across substance use disorders: a multi-level kernel density meta-analysis of resting-state functional connectivity studies. Addict. Biol. 27:e13200. doi: 10.1111/adb.13200, PMID: 35754101

[ref85] Thiebaut de Schotten M. Dell’Acqua F. Forkel S. Simmons A. Vergani F. Murphy D. G.. (2011). A lateralized brain network for visuo-spatial attention. Nat. Preced. 6:1. doi: 10.1038/npre.2011.5549.1 21926985

[ref86] Torosyan N. Mason K. Dahlbom M. Silverman D. H. Alzheimer’sDisease Neuroimaging Initiative. (2017). Value of FDG-PET scans of non-demented patients in predicting rates of future cognitive and functional decline. Eur. J. Nucl. Med. Mol. Imaging 44, 1355–1363. doi: 10.1007/s00259-017-3634-3, PMID: 28331953

[ref87] Touroutoglou A. Andreano J. Dickerson B. C. Barrett L. F. (2020). The tenacious brain: how the anterior mid-cingulate contributes to achieving goals. Cortex 123, 12–29. doi: 10.1016/j.cortex.2019.09.011, PMID: 31733343 PMC7381101

[ref88] Trick L. Kempton M. J. Williams S. C. Duka T. (2014). Impaired fear recognition and attentional set-shifting is associated with brain structural changes in alcoholic patients. Addict. Biol. 19, 1041–1054. doi: 10.1111/adb.12175, PMID: 25123156 PMC4282104

[ref89] Uddin L. Q. (2015). Salience processing and insular cortical function and dysfunction. Nat. Rev. Neurosci. 16, 55–61. doi: 10.1038/nrn3857, PMID: 25406711

[ref90] Uekermann J. Daum I. Schlebusch P. Wiebel B. Trenckmann U. (2003). Depression and cognitive functioning in alcoholism. Addiction 98, 1521–1529. doi: 10.1046/j.1360-0443.2003.00526.x, PMID: 14616178

[ref92] Volkow N. D. Wiers C. E. Shokri-Kojori E. Tomasi D. Wang G. J. Baler R. (2017). Neurochemical and metabolic effects of acute and chronic alcohol in the human brain: studies with positron emission tomography. Neuropharmacology 122, 175–188. doi: 10.1016/j.neuropharm.2017.01.012, PMID: 28108358

[ref93] Wang G. Li D. Y. Vance D. E. Li W. (2023). Alcohol use disorder as a risk factor for cognitive impairment. J. Alzheimers Dis. 94, 899–907. doi: 10.3233/JAD-230181, PMID: 37355899

[ref94] Weiland B. J. Sabbineni A. Calhoun V. D. Welsh R. C. Bryan A. D. Jung R. E.. (2014). Reduced left executive control network functional connectivity is associated with alcohol use disorders. Alcohol. Clin. Exp. Res. 38, 2445–2453. doi: 10.1111/acer.12505, PMID: 25257293 PMC4180110

[ref95] WHO (2014). Global status report on alcohol and health. Geneva: World Health Organization, 1–100.

[ref96] Wiers C. E. Gawron C. K. Gröpper S. Spengler S. Stuke H. Lindenmeyer J.. (2015). Decreased gray matter volume in inferior frontal gyrus is related to stop-signal task performance in alcohol-dependent patients. Psychiatry Res. Neuroimaging 233, 125–130. doi: 10.1016/j.pscychresns.2015.05.006, PMID: 26078198

[ref97] Xiao P. Dai Z. Zhong J. Zhu Y. Shi H. Pan P. (2015). Regional gray matter deficits in alcohol dependence: a meta-analysis of voxel-based morphometry studies. Drug Alcohol Depend. 153, 22–28. doi: 10.1016/j.drugalcdep.2015.05.030, PMID: 26072220

[ref98] Yang X. Tian F. Zhang H. Zeng J. Chen T. Wang S.. (2016). Cortical and subcortical gray matter shrinkage in alcohol-use disorders: a voxel-based meta-analysis. Neurosci. Biobehav. Rev. 66, 92–103. doi: 10.1016/j.neubiorev.2016.03.034, PMID: 27108216

[ref99] Zhu X. Cortes C. R. Mathur K. Tomasi D. Momenan R. (2017). Model-free functional connectivity and impulsivity correlates of alcohol dependence: a resting-state study. Addict. Biol. 22, 206–217. doi: 10.1111/adb.12272, PMID: 26040546 PMC4669235

