# Supplementary material for: Neural correlates of executive dysfunction in alcohol use disorder: preliminary evidence from 18F-FDG-PET
Source: Front Psychol. 2025 May 12;16:1568085. doi: 10.3389/fpsyg.2025.1568085 (PMC12104261; doi:10.3389/fpsyg.2025.1568085)
Supplement: Supplementary file 1 [file Data_Sheet_1.pdf]

SUPPLEMENTARY TABLES 1-7, SUPPLEMENTARY FIGURE 1

Supplementary table 1. Correlation Matrix.

	DS	IR	DR	IM-10	IM-30	TMT-A	TMT-B	TT	PF	AVR	CE	OF	CD	SD	CLD	PA
DS	1.000	.211	.100	.303	.011	-.171	-.188	-.300	.180	.152	.394	.157	.140	-.136	-.140	-.033
IR	.211	1.000	.787	.344	.171	-.369	-.448	.139	.088	.245	.118	.448	.151	.447	.365	.173
DR	.100	.787	1.000	.301	-.005	-.224	-.450	.344	.083	.151	.219	.333	.161	.363	.301	.047
IM-10	.303	.344	.301	1.000	.396	-.390	-.250	-.165	.089	.038	-.028	.252	-.002	.121	.089	.076
IM-30	.011	.171	-.005	.396	1.000	-.343	.100	-.084	.133	.008	-.170	.274	-.146	.299	.176	.279
TMT-A	-.171	-.369	-.224	-.390	-.343	1.000	.466	-.085	.119	-.104	-.024	-.525	-.104	-.152	-.283	-.005
TMT-B	-.188	-.448	-.450	-.250	.100	.466	1.000	-.164	.107	-.184	-.353	-.391	-.397	-.072	-.178	.092
TT	-.300	.139	.344	-.165	-.084	-.085	-.164	1.000	-.512	-.117	.226	.231	.037	.292	.087	-.044
PF	.180	.088	.083	.089	.133	.119	.107	-.512	1.000	.013	-.087	-.046	.106	-.080	.146	.107
AVR	.152	.245	.151	.038	.008	-.104	-.184	-.117	.013	1.000	.055	.207	.280	.127	.066	.295
CE	.394	.118	.219	-.028	-.170	-.024	-.353	.226	-.087	.055	1.000	.159	.403	.093	-.078	-.086
OF	.157	.448	.333	.252	.274	-.525	-.391	.231	-.046	.207	.159	1.000	.359	.479	.512	.353
CD	.140	.151	.161	-.002	-.146	-.104	-.397	.037	.106	.280	.403	.359	1.000	-.124	.096	-.086
SD	-.136	.447	.363	.121	.299	-.152	-.072	.292	-.080	.127	.093	.479	-.124	1.000	.469	.629
CLD	-.140	.365	.301	.089	.176	-.283	-.178	.087	.146	.066	-.078	.512	.096	.469	1.000	.576
PA	-.033	.173	.047	.076	.279	-.005	.092	-.044	.107	.295	-.086	.353	-.086	.629	.576	1.000

The table reports the cross-correlation coefficients among the 15 tests of the Brief Neuropsychological Examination (ENB2). DS = Digit span, IR = Immediate recall, DR= Delayed recall, IM-10 = Interference memory 10”, IM-30 = Interference memory 30”, TMT-A = Trial Making test A, TMT-B = Trial Making test B, TT = Token test, PF = Phonemic fluency, AVR = Abstract verbal reasoning, CE = cognitive estimation, OF = Overlapping figures, CD = copy drawing, SD = Spontaneous drawing, CLD = Clock drawing, PA = Praxis abilities.

Supplementary table 2. Communalities.

	Communalities
Digit span	.795
Immediate recall	.823
Delayed recall	.906
Interference memory 10’’	.682
Interference memory 30’’	.656
Trial Making test A	.792
Trial Making test B	.702
Token test	.809
Phonemic fluency	.754
Abstract verbal reasoning	.411
Cognitive estimation	.755
Overlapping figures	.752
Copy drawing	.743
Spontaneous drawing	.835
Clock drawing	.712
Praxis abilities	.856

For each variable of the Brief Neuropsychological Examination (ENB2), the table shows the proportion of variance explained by the retained components after the Varimax rotation.

Supplementary table 3. Rotated component matrix.

	Component					
	1	2	3	4	5	6
Digit span	**.916**					
Immediate recall	**.791**	.334				
Delayed recall	**.619**					-.387
Interference memory 10’’		**.936**				
Interference Memory 30’’		**.823**				
Trial Making test A			**-.779**	-.345		
Trial Making test B			**.711**			
Token test	.338		**.686**			
Phonemic fluency				**.839**		
Abstract verbal reasoning		-.466		**-.592**		
Cognitive estimation	.472		.439	**.496**		
Overlapping figures	.352			**.458**		
Copy drawing					**.844**	
Spontaneous drawing					**-.830**	
Clock drawing						**.791**
Praxis abilities				.315		**.716**

The table shows the correlations between the ENB variables and the estimated components after the Varimax rotation. Correlation coefficients < 0.3 are not reported.

Supplementary table 4. Neuro-cognitive performance.

a) Group comparison (two-sample t-test)								
Neuro-cognitive variables	mean HC	mean AUD	SD HC	SD AUD	DF	T-score/U*	p-value	FDR p-value
ENB2 global score	77.913	2.525	7.395	8.095	39	2.525	0.015	0.066
Digit span*	5.739	0.102	1.165	1.214	39	194.00	0.742	0.823
Immediate recall	12.826	1.728	4.570	4.270	39	1.728	0.0917	0.222
Delayed recall	18.826	0.940	5.041	5.131	39	0.940	0.352	0.666
Interference memory 10"	6.347	2.220	1.613	1.944	39	2.220	0.032	0.109
Interference memory 30"	6.521	0.627	2.013	2.233	39	0.627	0.533	0.823
**Trail Making test A**	29.130	-5.572	5.447	5.856	39	-5.572	<0.0001	**<0.0001**
Trail Making test B	89.782	-1.959	21.785	41.642	39	-1.959	0.057	0.162
Token test*	4.954	1.269	0.000	0.147	39	170.00	0.640	0.823
Phonemic fluency*	12.815	-0.295	3.433	3.279	39	113.000	0.800	0.823
Abstract verbal reasoning*	5.608	0.205	0.970	0.838	39	196.000	0.782	0.823
Cognitive estimation*	4.739	-0.105	0.460	0.540	39	197.000	0.802	0.823
**Overlapping figures**	31.173	3.294	5.514	5.605	39	3.294	0.002	**0.017**
Copy drawing*	1.652	1.153	0.383	0.572	39	177.000	0.438	0.745
Spontaneous drawing	1.739	1.037	0.323	0.540	39	1.037	0.306	0.650
Clock drawing*	8.295	1.426	2.348	2.462	39	102.500	0.009	0.055
Praxic abilities*	5.956	0.882	0.000	0.208	39	198.000	0.823	0.823
b) Group comparison controlling for age (ANCOVA)								
Neuro-cognitive variables	DF	F	p-value	FDR p-value				
**ENB2 global score**	1,38	6.169	**0.018**	**0.027**				
**Immediate recall**	1,38	2.845	**0.01**	**0.020**				
**Interference memory 10"**	1,38	4.700	**0.036**	**0.043**				
**Trail Making test A**	1,38	41.300	**<0.0001**	**<0.0001**				
**Overlapping figures**	1,38	10.440	**0.003**	**0.009**				
c) Group comparison controlling for education level (ANCOVA)								
Neuro-cognitive variables	DF	F	p-value	FDR p-value				
**ENB2 global score**	1,38	5.906	**0.020**	**0.033**				
Interference memory 10"	1,38	4.312	**0.045**	0.056				
**Trail Making test A**	1,38	28.39	**<0.0001**	**<0.0001**				
Trail Making test B	1,38	3.560	**0.067**	0.067				
**Overlapping figures**	1,38	9.584	**0.004**	**0.01**				

The top table sector reports the mean and SD of all ENB2 neuro-cognitive measures for the HC and the AUD groups separately, alongside the outcomes of group comparisons with two-sample t-tests. The middle and bottom sectors report the result of group comparisons while adjusting for age and education level, respectively, through ANCOVA. Asterisks indicate a non-normal distribution, while bold text highlights a statistically significant effect ($p<0.05$ corrected for multiple comparisons with False Discovery Rate (FDR). ENB: Brief Neuropsychological Examination (Mondini et al., 2011); DF: degrees of freedom.

Supplementary table 5. Total variance explained.

	A. Initial eigenvalues			B. Rotation sums of squared loadings		
Component	Total	% of variance	Cumulative %	Total	% of variance	Cumulative %
1	4.042	25.264	25.264	2.419	15.120	15.120
2	2.305	14.404	39.667	2.299	14.371	29.492
3	1.967	12.293	51.961	2.065	12.909	42.400
4	1.480	9.248	61.209	1.922	12.010	54.410
5	1.166	7.285	68.494	1.839	11.494	65.904
6	1.025	6.403	74.897	1.439	8.993	74.897
7	.927	5.794	80.691			
8	.686	4.287	84.978			
9	.530	3.310	88.288			
10	.465	2.906	91.193			
11	.350	2.191	93.384			
12	.311	1.946	95.329			
13	.283	1.767	97.097			
14	.202	1.262	98.359			
15	.142	.886	99.244			
16	.121	.756	100.000			

The table shows the distribution of the variance (eigenvalues, percentage of total variance accounted by each component, and the relative cumulative percentage) before (A) and after (B) a Varimax rotation. Based on the Kaiser-Guttman criterion (eigenvalue > 1), we retained six components explaining 74.89% of the total variance.

Supplementary table 6. Principal component analysis of neuro-cognitive data.

A: Principal component	B: Proportion of variance explained (cumulative proportion)	C: ENB2 tests	D: Loading coefficient
#1: Visuo-constructional abilities	15.12%	Praxis abilities	0.916
		Spontaneous drawing	0.791
		Clock drawing	0.619
#2: Verbal learning	14.37% (29.49%)	Delayed recall	0.936
		Immediate recall	0.823
#3: Basic-level executive functions	12.91% (42.4%)	Trail Making test A	-0.779
		Interference memory test 10”	0.711
		Interference memory test 30”	0.686
#4: High-level executive functions	12.01% (54.41%)	Copy drawing	0.839
		Trail Making test B	-0.592
		Overlapping figures	0.496
		Abstract verbal reasoning	0.458
#5: Language	11.49% (65.90%)	Phonemic fluency	0.844
		Token test	-0.830
#6: Estimation-related processes	8.99% (74.89%)	Digit span	0.791
		Cognitive estimation	0.716

The table reports the outcome of a principal component analysis performed on the scores from the Brief Neuropsychological Examination (ENB2; Mondini et al., 2011) for both AUD and HC groups. From left to right, the table shows: the first six principal components (with an eigenvalue greater than 1), which account for 74.89% of the overall variance in participants' performance across the 15 ENB2 tests (column A); the respective contribution, i.e., the cumulative proportion of explained variance, by each component (column B); the individual ENB2 tests that provide the largest contribution to each component (column C) and their loading coefficients (column D).

Supplementary table 7. Group comparisons of neuro-cognitive PCA factors.

a) Group comparison (two-sample t-test)								
Neuro-cognitive variables	mean HC	mean AUD	SD HC	SD AUD	DF	T-score	p-value	FDR p-value
PCA factor 01	0.083	-0.06	0.324	1.313	39	0.468	0.641	0.641
PCA factor 02	0.147	-0.115	1.003	1.003	39	0.832	0.410	0.49
**PCA factor 03**	0.534	-0.418	0.888	0.889	39	3.403	0.001	**0.009**
PCA factor 04	0.330	-0.258	0.817	1.068	39	1.933	0.060	0.181
PCA factor 05	-0.251	0.196	0.598	1.203	39	-1.444	0.156	0.313
PCA factor 06	-0.147	0.115	1.068	0.951	39	-0.832	0.410	0.49
b) Group comparison controlling for age (ANCOVA)								
Neuro-cognitive variables	DF	F	p-value	FDR p-value				
PCA factor 01	1,38	0.257	0.615	0.615				
PCA factor 02	1,38	0.577	0.452	0.542				
**PCA factor 03**	1,38	12.01	0.001	**0.006**				
PCA factor 04	1,38	3.634	0.064	0.192				
PCA factor 05	1,38	2.111	0.154	0.308				
PCA factor 06	1,38	0.627	0.433	0.542				
c) Group comparison controlling for education level (ANCOVA)								
Neuro-cognitive variables	DF	F	p-value	FDR p-value				
PCA factor 01	1,38	0.123	0.727	0.727				
PCA factor 02	1,38	0.533	0.470	0.564				
**PCA factor 03**	1,38	10.30	0.003	**0.018**				
PCA factor 04	1,38	4.002	0.053	0.159				
PCA factor 05	1,38	2.309	0.137	0.274				
PCA factor 06	1,38	0.745	0.394	0.564				
d) Group comparison controlling for age and education level (ANCOVA)								
Neuro-cognitive variables	DF	F	p-value	FDR p-value				
PCA factor 01	1,38	0.153	0.698	0.698				
PCA factor 02	1,38	0.418	0.522	0.626				
**PCA factor 03**	1,38	10.91	0.002	**0.012**				
PCA factor 04	1,38	3.889	0.056	0.168				
PCA factor 05	1,38	2.339	0.135	0.27				
PCA factor 06	1,38	0.668	0.419	0.626				

The top table sector reports the mean and SD of all six PCA factors for the HC and the AUD groups separately, alongside the outcomes of group comparisons with two-sample t-tests. The other sectors report the result of group comparisons while adjusting for age, education level, and both these demographic variables, respectively, through ANCOVA. Bold text highlights a statistically significant effect ($p<0.05$ corrected for multiple comparisons with False Discovery Rate (FDR). DF: degrees of freedom.

Supplementary figure 1. Control analyses with PVE-corrected FDG-PET images.

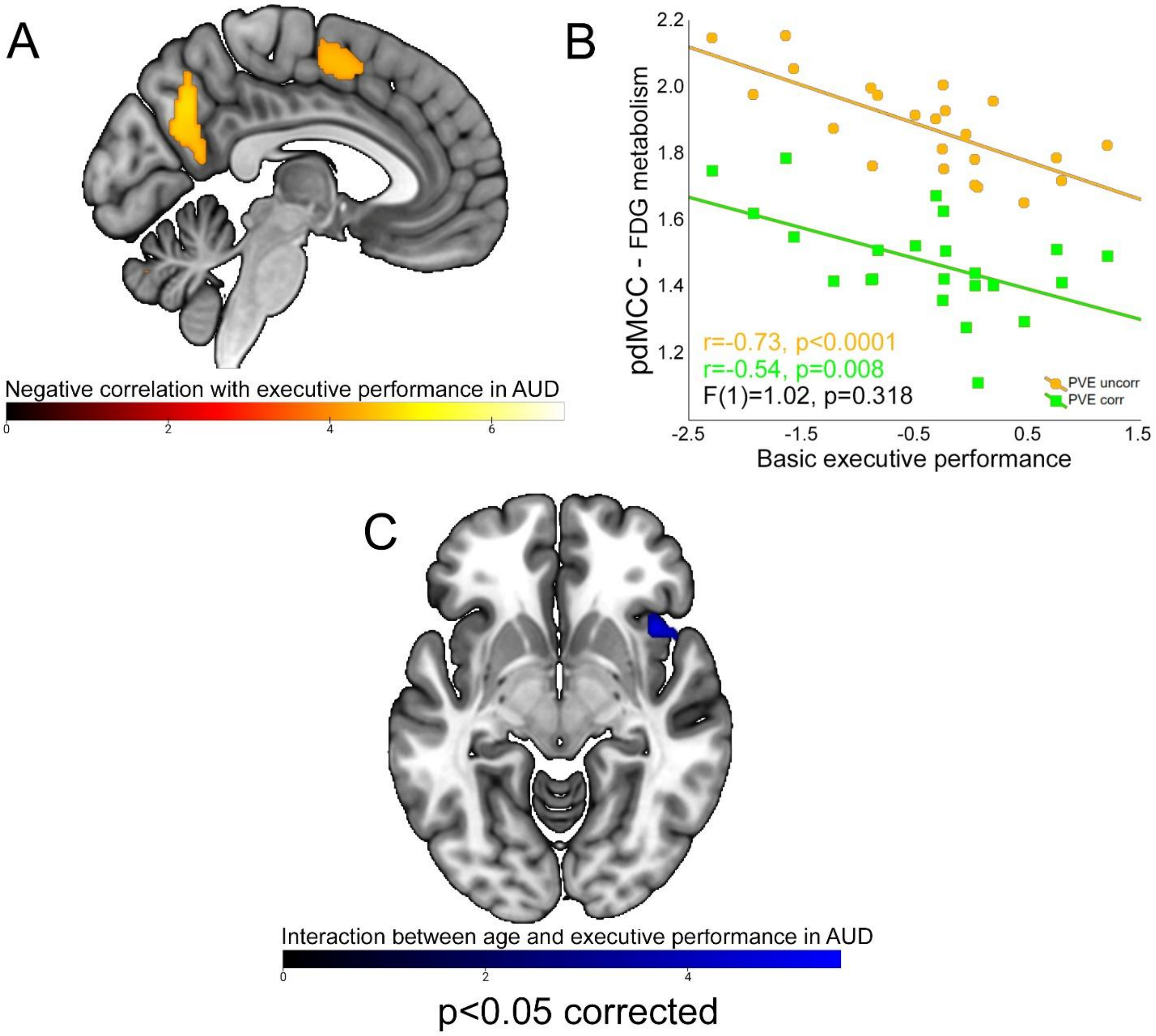

With different color-codes (as tracked by the respective colorbars), the figure depicts the regions in which brain metabolism was negatively correlated with executive performance in the AUD sample (yellow; A), and modulated by the interaction between age and executive performance (blue; C), after correcting for partial volume effects in AUD patients' FDG-PET brain images using their T1-weighted anatomical image and the modified Müller-Gärtner (mMG) approach as implemented in the PETPVE12 toolbox (Gonzalez-Escamilla et al., 2016). The scatterplot on the right shows that the negative correlation between executive performance and metabolism in the posterior dorsal midcingulate cortex holds both without (yellow) or with (green) the correction for partial volume effects.

References:

Mondini, S., Mapelli, D., Vestri, A., Arcara, G. & Bisiacchi, P. *Esame neuropsicologico breve. Una batteria di test per lo screening neuropsicologico*. (Raffaello Cortina, 2011)
